# Treatment of Long COVID or Post COVID syndrome: A Pharmacological approach

**DOI:** 10.3126/nje.v12i3.48532

**Published:** 2022-09-30

**Authors:** Indrajit Banerjee, Jared Robinson, Brijesh Sathian

**Affiliations:** 1, 2 Sir Seewoosagur Ramgoolam Medical College, Belle Rive, Mauritius; 3 Geriatrics and long term care Department, Rumailah Hospital, Hamad Medical Corporation, Doha, Qatar

## Background

The novel virus SARS-CoV-2 has plagued our earth for more than a quarter of a decade and is still currently a limiting factor in our daily lives. The global medical fraternity has overcome the numerous waves of the virus as well as the development and discovery of the various novel mutations and the relative challenges they posed [1]. Of late a new dilemma is beginning to incapacitate a large populous who have been infected by the virus, this condition being termed as post COVID syndrome or Long COVID [2]. Long COVID is becoming more prolific and common amongst those infected with COVID-19 and its development in patients does not delineate between those who have suffered from a symptomatic or an asymptomatic preliminary infection. The effect of this post COVID syndrome can be multisystemic in nature and can affect the daily cognitive abilities of the individual. It is therefore pertinent and of the upmost importance that therapeutic options against Long COVID are explored as the incidence of this condition is rising and will continue to do so in future [3,4].

### WHO definition of (clinical case definition) of post COVID-19 syndrome

The World Health Organizations case definition of Long COVID: “Occurs in individuals with a history of probable or confirmed SARS CoV-2 infection, usually 3 months from the onset of COVID- 19 with symptoms and that last for at least 2 months and cannot be explained by an alternative diagnosis. Common symptoms include fatigue, shortness of breath, cognitive dysfunction but also others and generally have an impact on everyday functioning. Symptoms may be new onset following initial recovery from an acute COVID-19 episode or persist from the initial illness. Symptoms may also fluctuate or relapse over time.” [5].

### Treatment of post COVID syndrome

Long COVID is a novel complication of COVID-19 and may manifest itself in a host of ways, due to it involving a plethora of the bodies systems. The treatment of Long COVID therefore is dynamic and multidisciplinary and is tailored to each individual case dependant on the involved systems and the patient’s relative signs and symptoms. In light of the absence of a specific and definitive treatment being available for Long COVID the management of chronic comorbidities and other concomitant diseases is essential throughout a patient’s therapy [6]. It is vital for treating physicians to diagnose the syndrome early and avoid further excessive and expansive investigations in search for an alternate diagnosis. A large proportion of individuals who suffer from the syndrome, reportedly seek selfcare and often resort to polypharmacy to gain some relief from the presenting foremost symptom [7] The treatment of Long COVID can thereby be divided into: symptomatic treatment, supportive treatment and rehabilitative treatment with self-monitoring [8].

### Symptomatic treatment

The symptomatic treatment in Long COVID will be dependant and specific to the presenting complaint and feature of the disease with which the patient presents, for example if the patient presents with fever, it can be treated with Paracetamol. Patients presenting with extreme fatigue can be prescribed Vitamin C. The necessary symptomatic treatment therefore varies on a patient-to-patient basis as well as a hospital-to-hospital basis dependant on the hospital protocol and local or national protocols in effect [9].

### Supportive treatment

Supportive treatment is a vital and major treatment modality employed in the therapy against Long COVID. Long COVID very frequently involves the neurological system with patients complaining of headaches, cognitive blunting “brain fog”, forgetfulness, depression, anxiety and insomnia. The current mainstay method for the treatment of these neurological symptoms is through supportive therapy and through cognitive behavioural therapy and counselling. The duty of the physician at the primary care level should not be underestimated and disregarded as the monitoring of these symptoms and most importantly the provision of reassurance to the patient is pertinent to their recovery and well-being. The primary care physician can also arrange subsequent referrals to the necessary psychiatric or psychological departments if the relative indications are present within a patient [10].

### Rehabilitative treatment with self-monitoring

The burden of the treatment of Long COVID does not simply fall onto the primary care physician but is a delicate interplay between self-monitoring, self-care by the patient and the treatment provided by the primary care physician. Self- monitoring and rehabilitative techniques have been prescribed and are being used in the regimen of therapy for post COVID breathlessness via daily serial pulse oximetry readings being recorded and logged by the patient in an at home setting. The pulse oximetry is successful in both patient reassurance and enables quicker recovery via the patient visualizing their healing process. Pulmonary rehabilitation and the treatment of chronic cough are instrumented through breathing exercises, which can vary from simple deep breathing techniques -breathing through pursed lips to specialist yoga breathing techniques; ultimately the goal being the reconditioning of the diaphragm and patient to take deeper breaths with breathing taking place through the nose, with no use of the accessory muscles [11].

### Emerging therapies and ongoing Clinical Trials

### Montelukast in the treatment of Long COVID respiratory symptoms

Montelukast is a leukotriene antagonist. The current E-speranza-COVID19 project is undertaking a randomized control trial which is currently in phase 3 testing. This trial is a double blinded and placebo-controlled drug trial which will determine whether Montelukast is a viable treatment option in patients suffering from Long COVID with respiratory symptoms. Currently 284 patients have been accepted into the ongoing trial with the treatment group receiving 10mg of Montelukast orally for 28 days. The trial is expected to be completed in August 2023 [12].

### Deupirfenidone in the treatment of Long COVID

Numerous clinical trials are underway for the use of the antifibrotic drugs viz. Deupirfenidone/ Pirfenidone. The trials are being conducted to determine whether Deupirfenidone is effective in the treatment of Post COVID pulmonary fibrosis. A case report from China describes the marked improvement in a 66-year-old female who was placed onto long term Pirfenidone therapy subsequently to being diagnosed with pulmonary fibrosis and the features of Long COVID. After 96 weeks of therapy, it was found that her dyspnoea level score improved from 4 to 2 and other parameters such as her five-minute walking distance, lung capacity and psychiatric scores improved [13].

### Vitamin C supplementation in the treatment of Long COVID

A study conducted by Vollbracht et al, which included 9 other clinical studies with a total of 720 participants concluded that 75% of the controlled trials and 80% of the observational studies performed on patients with Vitamin C supplementation recorded marked reductions in fatigue scores and reports. It was also noted that other parameters such as concentration, sleep hygiene and depression improved in individuals treated with Vitamin C. The rationale behind the use of Vitamin C in Post COVID syndrome would be for its immunomodulation and antioxidative properties. It is thus a recommended adjunct therapy in individuals suffering from long COVID [14].

### Nicotinamide riboside in the treatment of Long COVID

Nicotinamide riboside is part of the Vitamin B complex species and is currently under a randomised, phase 4 placebo controlled trial to determine its efficacy in the treatment of individuals with persistent cognitive and physical symptoms post COVID-19. A cohort of 100 participants have been enrolled into the trial with the trials estimated completion date to be the 31st of December 2023 [15].

### Leronlimab in the treatment of Long COVID

Leronlimab is an IgG4 monoclonal antibody which belongs to the species of CCR5 antagonists. It acts by immunomodulation and regulating the immune reactions within the body, it has been successfully used to treat individuals with acute COVID-19. A study conducted by Gaylis et al, on Leronlimab in Long COVID believe that the pathogenesis of the syndrome may be linked with CCR5 and its abnormal downregulation. The study concluded that the immuno-down regulation was normalized in patients treated with Leronlimab and with further investigation in the current ongoing trials (NCT0434365, NCT04347239, NCT04678830) may prove to be a treatment for long COVID [16].

### Melatonin in the treatment of Long COVID

Long COVID is believed to be due to a prolonged and protracted low grade inflammatory process. It has thus been hypothesized that a pharmacotherapeutic agent with anti-oxidative effects may be beneficial in the treatment of the syndrome. Melatonin is such a drug which activates NRF2 (nuclear factor erythroid-derived 2-like 2) which is believed to increase the formation of antioxidants on a cellular level. It is also noted that melatonin will have beneficial effects for the neurological sleep disturbances experienced in some patients with Long COVID and therefore Melatonin should be prescribed to patients whom will reap benefits therefrom [17].

### Adaptogens in the treatment of Long COVID

A randomized as well as a placebo-controlled trial (ADAPT-232) performed on 100 patients with the combination of (Rhodiola, Eleutherococcus, and Schisandra) adaptogens for a period of 14 days showed that the treatment group had marked improvements in renal function, physical performance capabilities and fatigue. Adaptogens are thus a possible and viable option for the treatment of Long COVID [18].

### Hyperbaric oxygen therapy in the treatment of Long COVID

Hyperbaric oxygen therapy otherwise known as (HBOT) is a new trend being used to effectively aid in the treatment of Post COVID fatigue. A study conducted by Robbins T, et al. on 10 patients who received 10 HBOT sessions with the duration of 105 mins each with 3 half an hour stints of 100% oxygen being administered per session has shown that HBOT produced a statistically significant improvement in the Chalder fatigue scale of the patients [19].

### Probiotic therapy in the treatment of Long COVID

Various postulations have pointed towards the disruption of the natural gastrointestinal microbiome to be the cause of Long COVID. If such a pathological interplay is responsible for the syndrome then the replacement and re-establishment of the intestinal microbiome is vital and of paramount importance. A randomized interventional, placebo controlled trial being performed on twenty participants with the probiotic (Omni-Biotic Pro Vi 5) is underway and is expected to be completed by the 31st of December 2022. The trial will determine whether the re-establishment of the intestinal microbial symbiosis will mitigate the Post COVID symptoms [20, 21].

## Conclusion

Currently no definitive treatment against Long COVID has been approved and the mainstay treatment is divided into Symptomatic, Supportive and Rehabilitative treatment with self-monitoring. The crux of the current treatment trends for Long COVID is that of a multidisciplinary approach with a great portion of the treatment implicating the patient to take an active role in their recovery, self-monitoring and treatment of the syndrome. Hyperbaric oxygen therapy as well as the use of adaptogens has shown promising results and is advisable to be added to the treatment regimen of patients to aid in decreasing fatigue in patients suffering from post COVID syndrome. The use of melatonin in patients suffering from insomnia and poor sleep hygiene is also advisable. Currently numerous pharmacotherapeutic agents ranging from Montelukast, Deupirfenidone, Nicotinamide riboside, Leronlimab, Adaptogens, Tocilizumab, Probiotics to HBOT are undergoing clinical trials. It is thus with hopeful expectancy that these drug trials will yield a viable definitive treatment for Long COVID.
